# Special Issue “Role of Exercises in Musculoskeletal Disorders—7th Edition”

**DOI:** 10.3390/jfmk11010049

**Published:** 2026-01-23

**Authors:** Giuseppe Musumeci

**Affiliations:** 1 Department of Biomedical and Biotechnological Sciences, Section of Anatomy, Histology and Movement Science, School of Medicine, University of Catania, 95123 Catania, Italy; giuseppe.musumeci@unict.it; 2 Research Center on Motor Activities (CRAM), University of Catania, 95123 Catania, Italy

## 1. Introduction

The seventh edition of the Special Issue “Role of Exercises in Musculoskeletal Disorders” further clarifies how exercise interventions can lead to measurable benefits across various musculoskeletal conditions, ages, and care settings. This collection contains a variety of experiments, including clinical cohorts, perioperative techniques, imaging optimization, and evidence synthesis, interrogating how we can better prescribe, deliver, and monitor interventions that restore function, reduce symptoms, and ameliorate the quality of life of people affected by musculoskeletal disorders. Several research topics were explored throughout these contributions, such as targeted loading and vibration, pragmatic metrics such as strength and walking capacity, how perioperative and rehabilitation details materially shape early function and efficiency, and imaging and elastography for assessment and follow-up. Exercise-based interventions are now positioned as core management for many musculoskeletal conditions because they can improve pain, physical function, and participation with comparatively low risk and broad applicability across different phenotypes and care settings [1]. In osteoarthritis, for example, the current guidelines explicitly frame physical activity and exercise as central to symptom management and function, emphasizing individualized goal setting, and the clinical reality that adapted physical acticity and tayoled exercise are considered as a first line of care for the disease management [2]. In musculoskeletal diseases like back pain, exercise is also recommended, with clinical practice guidelines defining it as a crucial intervention alongside electrotherapy, manual therapy, and medications [3]. In this context, by studying diverse themes and cohorts within exercise and musculoskeletal care, this seventh edition of the Special Issue advances our understanding in the fields of adapted kinesiology and sport–exercise science.

## 2. Overview of Published Articles

Operative technique and follow-up assessment are advanced by Papotto and colleagues (Contribution 1), who compared suprapatellar with infrapatellar intramedullary nailing for tibial shaft fractures in a large prospective cohort and simultaneously introduced the Catania Hospital Score as a functional outcome focused on trauma. Suprapatellar nailing improved positioning time, operative time, and fluoroscopy exposure, delivered better early clinician-rated function, and markedly reduced anterior knee pain, with similar union times and major complications to the infrapatellar approach. In clinical care for knee osteoarthritis, Coviello et al. (Contribution 2) compared single-injection cord-derived platelet-rich plasma with autologous PRP over a 12-month period. Both approaches produced meaningful improvements; cord-derived preparations offered earlier gains at 3–6 months, but outcomes converged by 9–12 months. A lower BMI and lower Kellgren–Lawrence grade predicted greater pain reduction, and safety profiles were similar. Returning to sport after hamstring injury, Kellis and colleagues (Contribution 3) examined footballers with prior biceps femoris strain using shear-wave elastography at multiple joint angles under passive and active conditions, alongside strength, cross-sectional area, and EMG. Compared with controls without previous injuries, the previously injured athletes demonstrated elevated active myofascial stiffness in semitendinosus and biceps femoris, reduced knee-flexion strength, and a smaller biceps femoris cross-sectional area, with similar EMG activity between groups. These findings argue for rehabilitation that targets fascia and muscle to normalize active mechanical behavior while addressing persistent strength and size deficits linked to re-injury risk. Diving into the Special Issue, Santilli et al. (Contribution 4) evaluated adults with non-calcific supraspinatus tendinopathy and delivered a short course of focused extracorporeal shockwave therapy and followed the effects for six months with shear-wave elastography and clinical scales. The authors noted that symptomatic tendons became thinner and stiffer at the elastography, while pain and functional scores improved; elastography changes correlated with clinical gains, positioning shear-wave elastography as a feasible biomarker for monitoring response. Basic and translational insights are represented by Cariati and colleagues (Contribution 5), who tested a 12-week whole-body vibration protocol in young, adult, and old mice. They observed increases in bone volume fraction and trabecular thickness and improved musculoskeletal health by the preservation of bone architecture and the up-regulation of FNDC5 and SIRT1 and down-regulation of NOX4, pointing to vibration as a candidate strategy to counter age-related skeletal decline. The authors conclude that autologous PRP remains the more pragmatic first-line option until larger randomized trials are available. Imaging optimization with direct relevance to musculoskeletal diagnostics was delivered by Wang et al. (Contribution 6), who refined lumbar oblique radiography in an Asian cohort using a customed positioning footboard and anthropometric stratification. A 35° angle obliquity yielded the best overall image quality, and waist-to-hip ratio outperformed BMI as a guide for angle selection; moreover, the positioning aid improved comfort and standardization. In patellofemoral pain syndrome, another study by Santilli and colleagues (Contribution 7) combined local muscle vibration to quadriceps vastus medialis obliquus with McConnell taping over three weeks, observing sustained six-month reductions in pain and improvements in KOOS. Responses were most pronounced in younger patients and in those with patellar laxation or subluxation on dynamic MRI. Lisboa et al. (Contribution 8) addressed the preoperative profiling in advanced knee osteoarthritis, by evaluating 122 patients who awaited total knee replacement, with quadriceps maximal voluntary isometric contraction, six-minute walk test performance, patient-reported outcomes, and radiographic severity. Quadriceps strength and walking capacity showed moderate associations with pain, function, and self-reported capacity, whereas radiographic grading did not meaningfully correlate with these measures that are patient centered. The authors highlighted that quadriceps strength and a 6-minute walking test were the most important functional variables that correlate with knee OA severity. Finally, Franceschi and colleagues (Contribution 9) conducted a systematic review comparing manual therapy plus therapeutic exercise with exercise alone for nonspecific musculoskeletal disorders related to the hip. From this qualitative synthesis, the authors concluded that there seems to be no difference in effectiveness between manual therapy combined with therapeutic exercise and therapeutic exercise alone in the treatment of patients with hip nonspecific musculoskeletal diseases.

## 3. Conclusions

The studies included in this Special Issue highlight that targeted exercise and load based strategies drive functional recovery, while adjuncts from vibration and shockwave therapy to taping, fascia sensitive rehabilitation, and optimized operative or imaging techniques can shape symptoms, efficiency, and early outcomes when matched to the specific phenotype of patients. In line with prior editions, the evidence confirm exercise as a fundamental intervention, complemented, and not substituted, by manual or device strategies tailored to patient goals and needs. Future studies should emphasize phenotype-guided trial designs, the durability of effects, cost-effectiveness, and integration of quantitative biomarkers to personalize rehabilitation.

## References

[1] Bannuru R.R., Osani M.C., Vaysbrot E.E., Arden N.K., Bennell K., Bierma-Zeinstra S.M.A., Kraus V.B., Lohmander L.S., Abbott J.H., Bhandari M. (2019). OARSI guidelines for the non-surgical management of knee, hip, and polyarticular osteoarthritis. Osteoarthr. Cartil..

[2] Gibbs A.J., Gray B., Wallis J.A., Taylor N.F., Kemp J.L., Hunter D.J., Barton C.J. (2023). Recommendations for the management of hip and knee osteoarthritis: A systematic review of clinical practice guidelines. Osteoarthr. Cartil..

[3] Zhou T., Salman D., McGregor A.H. (2024). Recent clinical practice guidelines for the management of low back pain: A global comparison. BMC Musculoskelet. Disord..

